# Differential Transcriptomes and Methylomes of Trophoblast Stem Cells From Naturally-Fertilized and Somatic Cell Nuclear-Transferred Embryos

**DOI:** 10.3389/fcell.2021.664178

**Published:** 2021-04-01

**Authors:** Jin Sun, Weisheng Zheng, Wenqiang Liu, Xiaochen Kou, Yanhong Zhao, Zehang Liang, Lu Wang, Zihao Zhang, Jing Xiao, Rui Gao, Shaorong Gao, Cizhong Jiang

**Affiliations:** ^1^Key Laboratory of Spine and Spinal Cord Injury Repair and Regeneration of Ministry of Education, Orthopaedic Department of Tongji Hospital, Shanghai Key Laboratory of Signaling and Disease Research, School of Life Sciences and Technology, Tongji University, Shanghai, China; ^2^Clinical and Translation Research Center of Shanghai First Maternity and Infant Hospital, Tongji University, Shanghai, China; ^3^Frontier Science Center for Stem Cell Research, Tongji University, Shanghai, China

**Keywords:** trophoblast stem cell, somatic cell nuclear transfer, transcriptome, DNA methylation, Scriptaid

## Abstract

Trophoblast stem cells (TSCs) are critical to mammalian embryogenesis by providing the cell source of the placenta. TSCs can be derived from trophoblast cells. However, the efficiency of TSC derivation from somatic cell nuclear transfer (NT) blastocysts is low. The regulatory mechanisms underlying transcription dynamics and epigenetic landscape remodeling during TSC derivation remain elusive. Here, we derived TSCs from the blastocysts by natural fertilization (NF), NT, and a histone deacetylase inhibitor Scriptaid-treated NT (SNT). Profiling of the transcriptomes across the stages of TSC derivation revealed that fibroblast growth factor 4 (FGF4) treatment resulted in many differentially expressed genes (DEGs) at outgrowth and initiated transcription program for TSC formation. We identified 75 transcription factors (TFs) that are continuously upregulated during NF TSC derivation, whose transcription profiles can infer the time course of NF not NT TSC derivation. Most DEGs in NT outgrowth are rescued in SNT outgrowth. The correct time course of SNT TSC derivation is inferred accordingly. Moreover, these TFs comprise an interaction network important to TSC stemness. Profiling of DNA methylation dynamics showed an extremely low level before FGF4 treatment and gradual increases afterward. FGF4 treatment results in a distinct DNA methylation remodeling process committed to TSC formation. We further identified 1,293 CpG islands (CGIs) whose DNA methylation difference is more than 0.25 during NF TSC derivation. The majority of these CGIs become highly methylated upon FGF4 treatment and remain in high levels. This may create a barrier for lineage commitment to restrict embryonic development, and ensure TSC formation. There exist hundreds of aberrantly methylated CGIs during NT TSC derivation, most of which are corrected during SNT TSC derivation. More than half of the aberrantly methylated CGIs before NT TSC formation are inherited from the donor genome. In contrast, the aberrantly methylated CGIs upon TSC formation are mainly from the highly methylated CGIs induced by FGF4 treatment. Functional annotation indicates that the aberrantly highly methylated CGIs play a role in repressing placenta development genes, etc., related to post-implantation development and maintaining TSC pluripotency. Collectively, our findings provide novel insights into the transcription dynamics, DNA methylation remodeling, and the role of FGF4 during TSC derivation.

## Introduction

Somatic cell nuclear transfer (NT) enables somatic nuclear to reprogram from the differentiated identity to a totipotent state, which allows the generation of cloned animals (Matoba and Zhang, 2018). It not only plays an important role in animal cloning but also shows great potential for human therapeutics. Despite its importance, the extremely low cloning efficiency has limited the development and application of NT (Yang et al., 2007). Recently, many studies found breakthroughs in improving NT efficiency, such as increasing histone acetylation levels by a histone deacetylase inhibitor (HDACi), for example, trichostatin A (TSA) (Kishigami et al., 2006; Rybouchkin et al., 2006; Zhao et al., 2010), reducing H3K9 and H3K4 methylation levels by overexpressing H3K9 and H3K4 demethylases (Chung et al., 2015; Hörmanseder et al., 2017), correcting abnormal *Xist* gene expression in donor nuclear or artificial zygotes (Inoue et al., 2010; Matoba et al., 2011). A recent study found that the transcription factor (TF) DUX can improve NT efficiency through mediating correct aberrant H3K9ac (Yang et al., 2021). By combining multiple epigenetic approaches, the blastocyst development rate reaches 95%, which is comparable to that of *in vitro* fertilized (IVF) embryos (Matoba et al., 2014; Liu et al., 2016). However, although the blastocyst rate of NT embryos largely improves from ∼1% to ∼20%, there exists a remarkable lag in pub rate of NT and IVF (Matoba et al., 2014; Liu et al., 2016), indicating that the post-implantation development barrier is still resistant, and these approaches function little on it.

The anatomical analysis reveals that placentomegaly is a common problem in NT post-implantation development (Palmieri et al., 2008). The cloned pups are frequently with large placentas, which also constitute expanded spongiotrophoblast layers, increased glycogen cells, restricted labyrinthine, and irregular borderlines between labyrinthine and spongiotrophoblast layers (Tanaka et al., 2001). It rescues abnormal placentas and improves the full-term development rate of NT by replacing NT trophectoderm (TE) with fertilized embryos by tetraploid complementation assay (Lin et al., 2011). Therefore, poor placental development of NT embryos may be a key factor contributing to the low rate of post-implantation development.

Many epigenetic reprogramming errors are related to abnormal placentas (Hemberger et al., 2020). Although HDACi treatment promotes the epigenetic reprogramming of donor nuclear and pre-implantation embryo development, it is helpless in improving post-implantation development (Gao R. et al., 2018). Recently, it has been reported that loss of maternal imprinting in NT placentas disrupts post-implantation development, and correcting their expression improves placenta development (Matoba et al., 2018; Inoue et al., 2020; Wang et al., 2020). Abnormal DNA methylation is an epigenetic barrier throughout the NT embryo development (Teperek and Miyamoto, 2013). Inhibiting aberrant DNA re-methylation by knockdown *Dnmt3a* and *Dnmt3b* ameliorates NT placentas (Gao R. et al., 2018), suggesting that aberrant re-methylation is another epigenetic cue for abnormal NT placenta.

Trophoblast stem cells (TSCs), located within the extraembryonic ectoderm (EXE) adjacent to the epiblast (EPI), are the precursors of the trophoblast cells in the placenta. TSC, which can be maintained from either blastocyst TE or EXE *in vitro*, is an invaluable model for placenta development, enabling researches on epigenetic regulation of self-renewal and differentiation (Oda et al., 2006). Fibroblast growth factor 4 (FGF4) signaling is a key to derivate TSCs *in vitro*; meanwhile, many TFs are essential including Cdx2, Eomes, Esrrb, Elf5, Tfap2c, and Sox2 (Ishiuchi et al., 2019). However, it still remains elusive how the regulatory networks organize and function in TSC derivation. Several groups established NT-TSCs and found that the transcriptome of NT-TSCs is globally similar to that of TSCs from natural fertilization (NF) embryos (Oda et al., 2009; Rielland et al., 2009; Soares and Asanoma, 2009). Besides, the targeted sequencing analysis revealed that there is loss of imprinting in NT-TSCs (Hirose et al., 2018). The efficiency of isolating NT-TSC colonies from NT blastocysts is predicted to be low compared with that from NF blastocysts (Oda et al., 2010). Whether HDACi treatment can improve TSC derivation as it improves NT cloning is unknown. The global DNA methylation remodeling has not been reported, either. Answers to these questions will facilitate TSC derivation and its application in stem cell research, especially in the study of placenta development.

Therefore, in this study, we collected cell samples at five time points during the derivation of TSCs from NF, NT, and NT embryos with HDACi (Scriptaid) treatment (SNT). We investigated the changes in gene expressions and DNA methylation during TSC derivation, and the difference between TSC derivation from NF, NT, and SNT blastocysts. Our study identified a set of 75 TFs whose transcription profiles can infer the time course of TSC derivation. Moreover, a tight interaction network containing the TF ZFP281 is important to TSC formation. Scriptaid treatment rescues the expression of these TFs. FGF4 treatment increases DNA methylation in outgrowth directing progress to TSC formation. Interestingly, the specifically highly methylated CpG islands (CGIs) in the outgrowth derived from inner cell mass (ICM) cultured with FGF4 become aberrantly highly methylated (AHM) in NT and SNT TSCs. This suggests that high methylation induced by FGF4 is critical to TSC derivation and maintenance. These findings shed new light on the transcription and DNA methylation reprograming and the concomitant regulatory mechanisms underlying TSC formation and maintenance.

## Results

### Generation of Trophoblast Stem Cells From Natural Fertilization, Nuclear Transfer, and Scriptaid-Treated NT Embryos and Profiling of the Transcriptomes

**FIGURE 1:**
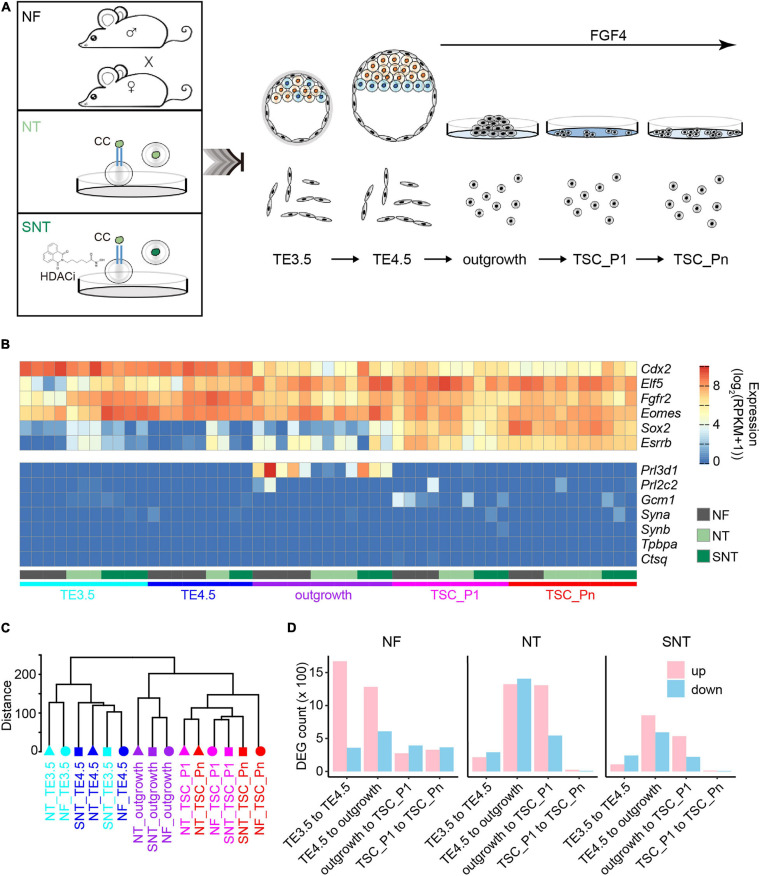
Trophoblast stem cell (TSC) derivation and profiling of the transcriptomes. **(A)** Schematic diagram for TSC derivation from natural fertilization (NF), nuclear transfer (NT), and Scriptaid-treated NT (SNT) blastocysts, which NF represents the embryos from natural fertilization; NT, somatic cell nuclear transfer; SNT, NT with histone deacetylase (HDAC) inhibitor (Scriptaid) treatment. TSC_P1: TSC formation (passage 1). TSC_Pn are TSCs cultured for three to four passages (see section “Materials and Methods” for details). **(B)** A heat map showing the normalized expression of pluripotency genes (top), and differentiation genes (bottom). Of note, the marker genes for the different trophoblast subtypes are *Gcm1*, labyrinthine trophoblast marker; *Tpbpa*, spongiotrophoblast marker; *Prl3b1*, giant cell marker. **(C)** A dendrogram showing clustering of gene expressions of all samples. The replicates of each sample are merged using the mean values. **(D)** The statistics of the differentially expressed genes (DEGs) between adjacent stages during TSC derivation. Red, upregulated DEGs; blue, downregulated DEGs.

We first generated TSC lines from the embryos produced by NF, NT, and NT with HDAC inhibitor (Scriptaid) treatment (SNT) (Figure 1A). The E3.5 blastocysts were collected and continued to expand *in vitro* until the zona pellucida was broken, that is, E4.5 blastocysts. The TE of E3.5 blastocysts (TE3.5) and E4.5 blastocysts (TE4.5) were collected, respectively. The TE4.5 were cultivated *in vitro* and attached to form an outgrowth on the second day with exogenous FGF4 supplement to derive TSCs. The first appeared TSC colonies were designated as passage-1 TSC (termed as TSC_P1). TSC_P1 were cultured for three to four passages and became the virtually immortal TSC lines (termed as TSC_Pn) without significant differentiation. (see section “Materials and Methods” for details.) Collectively, there are five samples TE3.5, TE4.5, outgrowth, TSC_P1, and TSC_Pn derived from NF, NT, and SNT embryos. Of note, only NF TE3.5 is developed *in vivo*, others are cultivated *in vitro*. These samples were used to explore the changes in gene expressions and DNA methylation, their difference between NF, NT, and SNT approaches, and potential rescue mechanisms of HDAC inhibitor.

We next profiled the gene expressions using RNA-seq with two to five biological replicates for each sample with high reproducibility (Supplementary Figure 1A). The pluripotency genes, including TSC marker genes *Fgfr2*, *Sox2*, and *Esrrb*, are highly expressed. Contrarily, the differentiation genes, including the marker genes for different trophoblast subtypes *Gcm1*, *Tpbpa*, and *Prl3b1*, are lowly expressed or silenced throughout the TSC generation (Figure 1B). This is consistent with the previous results (Ji et al., 2013; Latos and Hemberger, 2014). This confirms the pluripotency of the TSCs derived from NF, NT, and SNT embryos.

The unsupervised hierarchical clustering analysis of the transcriptome data results in two major groups: TE3.5 and TE4.5, outgrowth, and TSCs for NF, NT, and SNT samples, respectively (Supplementary Figures 1B–D). This pattern holds when NF, NT, and SNT samples are combined (Figure 1C). Notably, outgrowth is grouped with TSCs rather than TE. This indicates that FGF4 treatment initiates the transcription program toward TSCs.

To understand the changes in gene expressions during the TSC derivation, we identified the differentially expressed genes (DEGs) between the adjacent stages. The results show that the number of DEGs from TE3.5 to TE4.5 and from TE4.5 to outgrowth is much more than that between later adjacent stages during NF TSC generation (Figure 1D). In contrast, the number of DEGs from TE4.5 to outgrowth and from outgrowth to TSC_P1 is the largest during both NT and SNT TSC generation. However, the changes in gene expressions during SNT TSC generation have a lesser extent (Figure 1D). This indicates the rescue effect of the HDACi Scriptaid. The prominent changes in gene expressions from TE3.5 to TE4.5 only during NF TSC generation are likely due to the TE4.5 cultured *in vitro* from TE3.5 *in vivo*. The dramatic changes in gene expression after TE4.5 during the three types of TSC generation are likely due to FGF4 treatment. These different transcriptome dynamics suggest the distinct transcription programing during the three types of TSC generation. To understand the functions of these DEGs, we collected specifically expressed genes of tissues and cell lines from paGenBase (Pan et al., 2013) and performed gene ontology (GO) analysis (Zhou et al., 2019). The results show that the overall trend of transcription programing toward TSC derivation is to activate placenta genes and to inactivate blastocyst genes upon FGF4 treatment. Once TSC is formed, both placenta and blastocyst genes are inactivated to maintain pluripotency. However, both placenta and blastocyst genes remain downregulated only during NF TSC passaging (Supplementary Figure 1E). This partially explains the higher quality of NF TSCs.

### The Key Transcription Factors Reveal the Derivation Progression of Natural Fertilization Trophoblast Stem Cells

**FIGURE 2:**
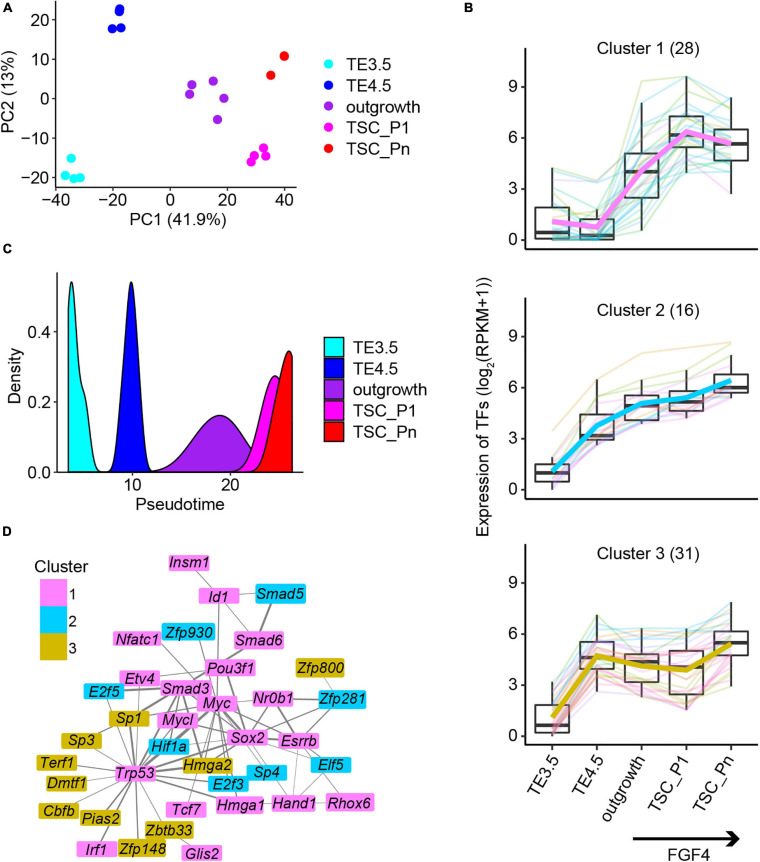
The key transcription factors critical to the derivation of NF TSCs. **(A)** Principal component analysis (PCA) of the transcriptomic data of NF samples. **(B)** Expression patterns of the 75 transcription factors in Clusters 1–3 (Supplementary Figure 2B). **(C)** The pseudotime of NF TSC derivation inferred from the transcriptomes of the 75 transcription factors (TFs) in **(B)**. **(D)** Protein–protein interaction (PPI) networks of the 75 TFs in **(B)**. The thickness of the lines represents the PPI scores, and the color represents the gene cluster in Supplementary Figure 2B.

The principal component analysis (PCA) of the transcriptomes recapitulated the time course of TSC derivation from TE3.5 (Figure 2A). To further identify the key dynamically expressed genes critical to TSC derivation, we collected the genes in the top 5% of absolute principal component loadings (Supplementary Figure 2A). The unsupervised hierarchical clustering of these dynamically expressed genes resulted in six clusters (Supplementary Figure 2B). Clusters 1–3 are continuously upregulated from TE4.5 or outgrowth (termed as Pro-genes). Cluster 4 is continuously downregulated from outgrowth (termed as Down-genes). Clusters 5 and 6 are transiently down- and upregulated, respectively (together termed as transient genes) (Supplementary Figure 2B). The functional annotation of these genes revealed that Clusters 1–3 are enriched for GO terms promotional to TSC derivation (Supplementary Figure 2C). Particularly, Cluster 1 is enriched for embryonic placenta development, epithelial cell differentiation, signaling pathways, regulating pluripotency of stem cells, and mesenchyme development. Moreover, many TFs in Custer 1 are direct targets of FGF4/ERK signaling pathway (Adachi et al., 2013; Latos et al., 2015). This together consists of the upregulation upon FGF4 treatment (Supplementary Figure 2B). Clusters 2 and 3 are mainly enriched for cell cycle-related GO terms (Supplementary Figure 2C), which are important to the acquisition of pluripotency.

Transcription factors regulate gene expression and control cellular function and cell fate. Thus, we identified a total of 106 TFs in the six clusters of genes (Supplementary Figure 2B). The 75 TFs from the Pro-genes (Clusters 1–3) (termed Pro-TFs) show a continuously upregulated expression (Figure 2B). Surprisingly, we inferred the pseudotime only from the 75 TFs’ transcriptomic data, which exactly matches the time course of TSC derivation (Figure 2C). However, when we used random 75 TFs to perform the same analysis, we failed to obtain the trajectory to TSC derivation (Supplementary Figure 2D). This suggests that these 75 TFs are informative to explore the progression of TSC derivation. Then, we attempted to identify interaction networks between these TFs. The results showed a tight interaction network consisting of 37 TFs (Figure 2D). The network was partially shared by the pluripotency network in ESCs. The TFs in network ESRRB, SOX2, and NR0B1 were reported to confer the pluripotency of ESCs (Adachi et al., 2013; Gao H. et al., 2018). However, the TFs ZFP281 and ELF5 are specifically related to TSC stemness (Gao H. et al., 2018; Ishiuchi et al., 2019).

### Scriptaid Treatment Largely Rescues Abnormal Gene Expressions in the Derivation of Scriptaid-Treated Nuclear Transfer Trophoblast Stem Cells

**FIGURE 3:**
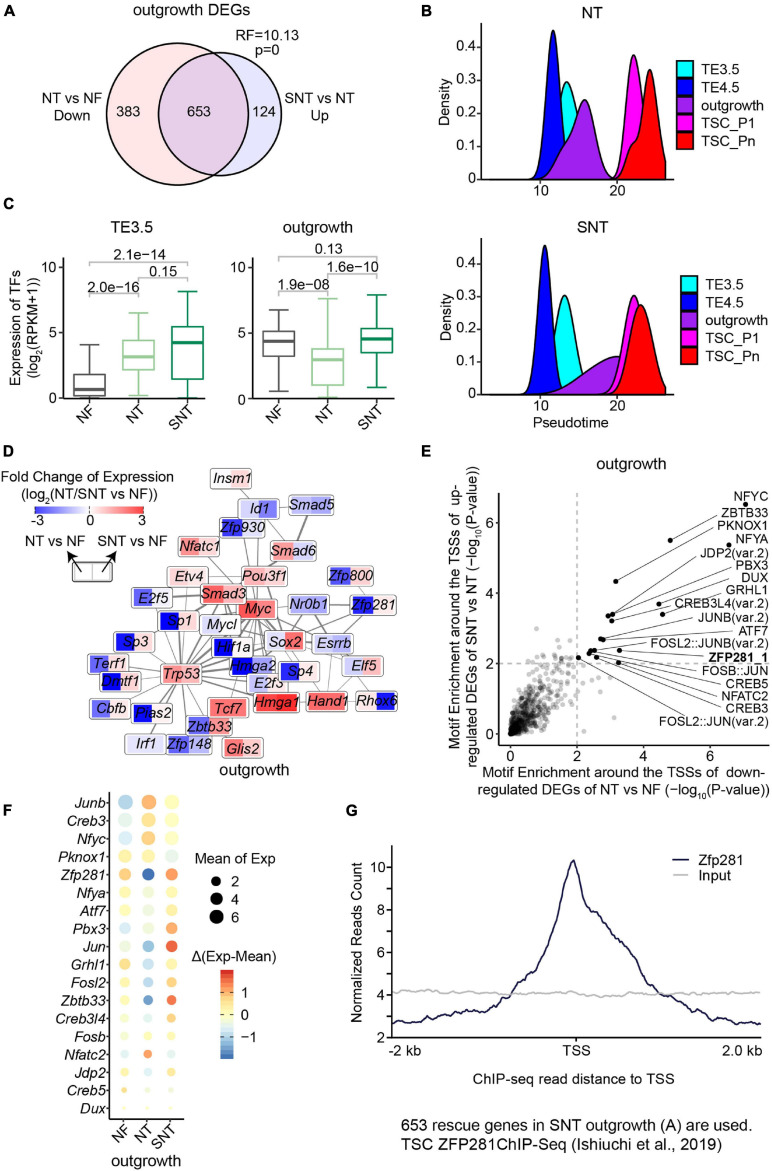
Downregulated genes in NT outgrowth are largely rescued in SNT outgrowth. **(A)** Venn diagrams showing the intersection between the downregulated genes in NT outgrowth compared with NF outgrowth and the upregulated gene SNT outgrowth compared with NT outgrowth. Representation factor (RF) was calculated using real observation/expected observation. The statistical analysis is hypergeometric test with all expressed genes at outgrowth as background. The intersection genes are defined as the rescued genes. **(B)** The pseudotime of NT (top) or SNT (bottom) TSC derivation inferred from the transcriptomes of the 75 TFs in Clusters 1–3 (Supplementary Figure 3D). **(C)** Boxplots showing the expression of the 75 TFs in Clusters 1–3. Paired *t*-test is performed for statistical comparisons, and “holm” is used for adjusting *p*-values. **(D)** Expression changes of the component TFs in the PPI network (Figure 2D) in NT outgrowth are recued in SNT outgrowth. Each box is divided into two parts. Left part represents the expression difference between NT and NF outgrowth. Right part represents the expression difference between SNT and NF outgrowth. Color scale bar indicates the fold change of gene expression between NT/SNT and NF in the form of Log2. Red indicates increased expression. Blue indicates decreased expression. **(E)** Scatterplot showing the significance of the motif enrichment in the proximal TSS regions of the two gene sets in **(A)**. **(F)** Bubble plot showing gene expressions of the TFs whose motifs are identified in **(E)**. The size of the bubble indicates the mean gene expression at NF, NT, and SNT outgrowths. The color indicates the difference from the mean. **(G)** ZFP281 ChIP-seq signal distribution around transcript start sites (TSSs) in the rescued genes [the intersection part in **(A)**]. TSC ZFP281 ChIP-seq data is from GSE111824 (Ishiuchi et al., 2019). The dark blue line indicates ZFP281 ChIP-seq data, while the gray line indicates the input signal.

The PCA of all the transcriptomic data in the derivation process of TSCs shows that the NF, NT, and SNT TE3.5 samples are separate from one another. In contrast, NF outgrowth samples are separate from NT outgrowth but close to SNT outgrowth (Supplementary Figure 3A). Consistently, the number of DEGs between NT/SNT and NF TE3.5 samples is much larger than that between NT/SNT and NF outgrowth samples. Moreover, most of DEGs between NT/SNT and NF TE3.5 samples are common, while most of the DEGs between NT/SNT and NF outgrowth samples are different (Supplementary Figures 3B,C). Interestingly, most of the downregulated DEGs between NT and NF outgrowth samples are rescued in SNT samples (Figure 3A). These findings suggest that the TE3.5 transcription program is very different between the NF, NT, and SNT approaches. The outgrowth transcription program is close between the NF and SNT approach.

We next examined the expression patterns in NT and SNT TSC derivation of the key dynamically expressed genes identified in NF TSC derivation. The results show that the expression patterns of gene Clusters 1–3 in NT and SNT TSC derivation are distinct from NF TSC derivation (Supplementary Figure 3D). The expressions of TFs in gene Clusters 1–3 succeed in inferring the trajectory to NF TSC derivation (Figure 2C). However, the similar analysis in NT and SNT TSC derivation led to incorrect time course (Figure 3B). Compared with NF TSC derivation, TE3.5 is erroneously put after TE4.5 in both NT and SNT TSC derivation. Besides, outgrowth is a narrow peak not overlapping TSC_P1, indicating a gap between the transition from outgrowth to TSC formation. Conversely, like NF outgrowth, SNT outgrowth is a broad peak overlapping with TSC_P1, suggesting that certain transcription programs for TSC formation have been initiated in outgrowth (Figures 2C, 3B). Further analysis reveals that the expressions of TFs in Cluster 1 are significantly increased in NT TE3.5 but rescued in SNT TE3.5. However, the expressions of TFs in Clusters 2 and 3 are significantly increased in both NT and SNT TE3.5 (Supplementary Figure 3E). Due to the number of TFs in Clusters 2 and 3 that is much larger than that in Cluster 1, the overall expressions of TFs in Clusters 1–3 are significantly increased in both NT and SNT TE3.5 (Figure 3C). Therefore, TE3.5 is incorrectly put in the inferred pseudotime of NT and SNT TSC derivation. Conversely, the expressions of TFs in Cluster 1 remain unchanged in both NT and SNT outgrowth. However, the expressions of TFs in Clusters 2 and 3 are significantly decreased in NT but rescued in SNT outgrowth (Supplementary Figure 3E). Due to the number of TFs in Clusters 2 and 3 that is much larger than that in Cluster 1, the overall expressions of TFs in Clusters 1–3 are significantly decreased in NT but rescued in SNT outgrowth (Figure 3C). Thus, SNT outgrowth has a pattern similar to NF outgrowth in the inferred pseudotime of TSC derivation, while NT outgrowth has an aberrant pattern (Figure 3B). Intriguingly, most of the component TFs in the interaction network are downregulated in NT but partially or fully rescued in SNT (Figure 3D). These findings further indicate that the correct transcription programs of these key TFs play an important role in TSC derivation.

### ZFP281 Is Critical to Outgrowth Formation

We observed that most of the downregulated DEGs between NT and NF outgrowth are rescued in SNT outgrowth (Figure 3A). This likely contributes to the aberrant pattern of NT outgrowth in the pseudotime of TSC derivation (Figure 3B). Thus, it is important to identify the potential upregulators that rescue the DEGs. To this end, we analyzed motif enrichment in the promoter regions of the downregulated DEGs between NT and NF outgrowth that are rescued in SNT outgrowth. The results identified 18 motifs significantly enriched in both sets of DEGs (Figure 3E). Examination of the expressions of the TFs corresponding to the 18 motifs shows that TF *ZFP281* is significantly downregulated in NT outgrowth but rescued in SNT outgrowth (Figure 3F). Moreover, *Zfp281* is downregulated only in NT outgrowth in the TSC derivation (Supplementary Figure 4A). Previous studies had reported that *Zfp281* is essential for early placenta development and TSC maintaining, and it interacts with MLL/COMPASS subunits to bind to the promoters of target genes to activate transcription (Ishiuchi et al., 2019). Therefore, we explored ZFP281 ChIP-seq signals around the transcription start sites (TSSs) of the 653 rescued genes in outgrowth defined in Figure 3A. The results show enrichment of ZFP281 ChIP-seq signals around the TSSs, indicating ZFP281 binding in the promoter regions of the rescued genes (Figure 3G). Consistently, a set of key dynamically changed TFs in Clusters 1–3 are significantly downregulated in NT outgrowth, whose promoter regions are bound by ZFP281 (Supplementary Figures 4B,C). Downregulation of *Zfp281* expression in NT outgrowth also contributes to the reduced interaction in the network (Figure 3D). Collectively, ZFP281 is a core factor critical to outgrowth formation.

### DNA Methylation Dynamics in CpG Islands During the Derivation of Trophoblast Stem Cells

**FIGURE 4:**
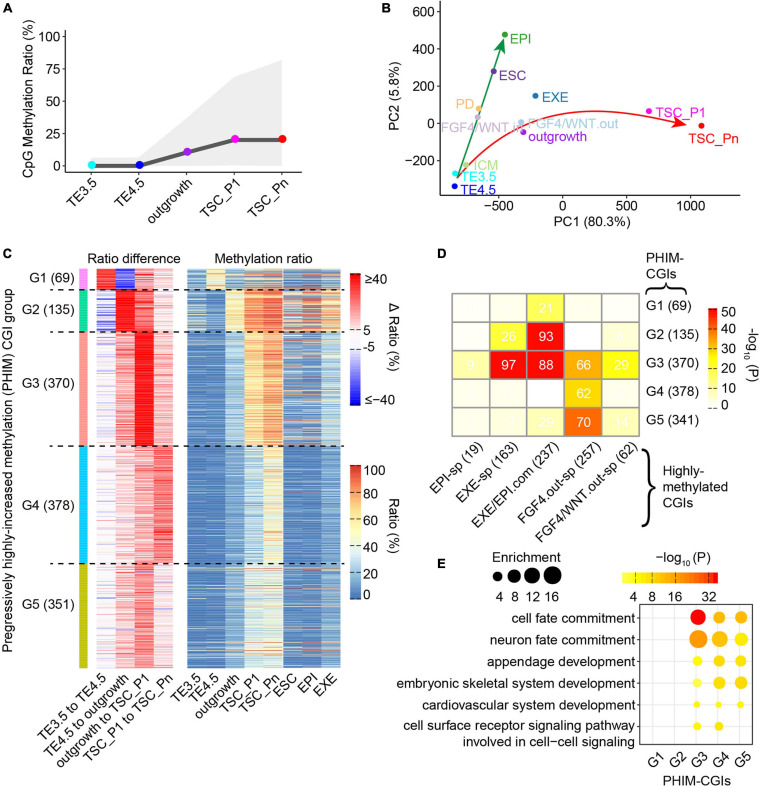
Fibroblast growth factor 4 (FGF4) functions in methylation remodeling during TSC derivation. **(A)** CpG methylation levels at each stage of TSC derivation. The line indicates the medians. The shaded area represents the 25th to 75th percentiles. **(B)** Principal component analysis (PCA) of CpG island (CGI) methylation. The reduced representation bisulfite sequencing (RRBS) data of perturbingly cultured inner cell mass (ICM) are from GSE98963 (Smith et al., 2017). FGF4/WNT.in and FGF4/WNT.out represent the internal and outer part of the outgrowth derived from ICM cultured in the basal media supplemented with FGF4 and WNT agonist CHIR99021, respectively. FGF4 and CHIR99021 do not reach FGF4/WNT.in; FGF4/WNT.out, the outer layer of the outgrowth, responded to FGF4 and CHIR99021. PD represents ICM cultured in the basal media supplemented with MAPKK or MEK inhibitor PD0325901 and FGF4. The RRBS data of ESCs are from GSE47343 (Guo et al., 2013). **(C)** Heat maps showing K-mean clustering of the PHIM-CGIs during TSC derivation, whose DNA methylation level difference between adjacent stages is larger than 25%. Left heatmap showing CGI DNA methylation level difference between adjacent stages. Right heatmap showing CGI DNA methylation levels in each sample. **(D)** A heat map showing the intersection between the PHIM-CGIs **(C)** and the highly methylated CGIs (>0.25). Filled colors indicate the significance of the intersection (hypergeometric test). Numbers indicate CGI count. “EXE/EPI-com” denotes the CGIs that are highly methylated in both EXE and EPI. “EXE-specific” and “EPI-specific” denote the CGIs that are highly methylated specifically in EXE and EPI, respectively. “FGF4.out-sp” denotes the CGIs that are highly methylated in ICM treated with FGF4 but not in EXE and EPI. “FGF4/WNT.out-sp” denotes the CGIs that are highly methylated in outgrowth outer layers derived from ICM treated with FGF4 and WNT agonist CHIR99021 but not in EXE and EPI. **(E)** Bubble plots showing the gene ontology (GO) terms significantly enriched in the PHIM-CGIs **(C)**.

DNA methylation is important in early trophoblast development (Branco et al., 2016), and it had been reported that HDACi treatment accelerates the DNA methylation reprogramming of SCNT (Jin et al., 2017). To understand the dynamics and the role of DNA methylation during TSC derivation, we took advantage of reduced representation bisulfite sequencing (RRBS) to profile the DNA methylomes across TSC derivation. A previous study identified two groups of sites: methylated in TSCs not in ESCs, unmethylated in both TSCs and ESCs (Oda et al., 2009). Consistently, the TSC-specific methylation sites are gradually methylated and the unmethylated sites remain unmethylated during TSC derivation (Supplementary Figure 5A). The DNA methylomes show that TE3.5 and TE4.5 are unmethylated, while the global DNA methylation levels increase from outgrowth to TSC (Figure 4A). RRBS mainly covers the majority of CGIs, especially DNA methylation in the promoters (Meissner et al., 2008). We therefore examined the dynamics of DNA methylation in CGIs during TSC derivation. The results show that the majority of CGIs (∼75%) exhibited low methylation across TSC derivation. A small fraction of CGIs become highly methylated in outgrowth. More CGIs (∼10%) are highly methylated upon TSC formation (Supplementary Figure 5B). Approximately 70% of mouse gene promoters are associated with a CGI (Deaton and Bird, 2011). High-CpG-density promoters (HCPs) remain unmethylated or lowly methylated. Low-CpG-density promoters (LCPs) remain lowly methylated at TE3.5 and TE4.5 but gradually methylated from outgrowth. Intermediate-CpG-density promoters (ICPs) have DNA methylation dynamics between that of HCPs and LCPs (Supplementary Figure 5C).

It was reported that dense CpG methylation in CGIs represses the nearby gene transcription (Deaton and Bird, 2011). Our results show that CGI methylation has weak correlation with gene expression at TE3.5 and TE4.5 due to global DNA demethylation process in pre-implantation embryo development (Messerschmidt et al., 2014), and the regulation of CGI methylation strengthens as DNA methylation is gradually established from outgrowth (Supplementary Figures 5D,E). Of note, DNA methylation level is very low and unchanged in the promoters of the Pro-genes during TSC derivation. However, the Pro-genes are continuously upregulated from TE4.5 or outgrowth (Supplementary Figures 2B,5F). This indicates that the DNA methylation has no correlation with the transcription of the Pro-genes.

### FGF4 Contributes to High Methylation in CGIs During the Derivation of Trophoblast Stem Cells

To understand the potential role of FGF4 in DNA methylation dynamics during TSC derivation, we performed the PCA of DNA methylation in CGIs. The results show the divergent trajectories of embryonic and extraembryonic development as in previous studies (Senner et al., 2012; Figure 4B). Specifically, the early stages (TE3.5, TE4.5, and ICM) of embryonic and extraembryonic development are clustered together. However, when outgrowth was derived from ICM cultured with FGF4 and WNT agonist CHIR99021, the outer layers (termed as FGF4/WNT.out) and the inner parts (termed as FGF4/WNT.in) of this outgrowth have differential methylome profiles. FGF4/WNT.out is clustered with our outgrowth and follows the TSC derivation path. Conversely, FGF4/WNT.in is clustered with PD and follows the path to EPI (Figure 4B). PD is the outgrowth derived from ICM cultured with FGF4 plus MAPKK or MEK inhibitor PD0325901 that blocks the downstream pathways of FGF4. Of note, FGF4 and CHIR99021 can reach the outer layers of outgrowth. That is, FGF4/WNT.out is similar to our outgrowth. In contrast, FGF4 and CHIR99021 cannot reach the inner layers of outgrowth. These findings suggest that FGF4 coordinates with WNT in remodeling and establishing a unique DNA methylation landscape in TSCs. This is consistent with the previous report that FGF4 and WNT are the main signaling pathways, involved in early placenta development and TSC derivation (El-Hashash et al., 2010; Lanner and Rossant, 2010).

We next identified 1,293 CGIs whose max difference of the DNA methylation ratio is larger than 25% during TSC derivation (termed as progressively highly increased methylation CGIs, PHIM-CGIs), which were clustered into five groups. Basically, DNA methylation levels increase in each group of CGIs at a certain stage of TSC derivation. Interestingly, DNA methylation levels in the Group 1 of PHIM-CGIs transiently increase at TE4.5 and decrease back to the original levels of TE3.5 at outgrowth. Notably, these 1,293 CGIs remain a high level of DNA methylation in TSCs (Figure 4C). This indicates that high DNA methylation levels in these CGIs are critical to TSCs. We further identified all highly methylated CGIs (>0.25) across TSC derivation and in EXE, EPI, and ESCs. The results manifest that the majority of these CGIs are *de novo* highly methylated upon TSC formation. Moreover, these CGIs include all highly methylated CGIs in EXE, EPI, and ESCs. Of note, most of these CGIs have a higher methylation ratio in TSCs than in EXE, EPI, and ESCs (Supplementary Figure 5G). Collectively, high methylation levels in these CGIs are important to TSC and make TSCs different from EXE, EPI, and ESCs.

Comparing the PHIM-CGIs with the highly methylated CGIs, we found that EXE-specific highly methylated CGIs are significantly enriched in the Group 3 of PHIM-CGIs. Similarly, EXE and EPI common highly methylated CGIs are significantly enriched in Groups 2 and 3 (Figure 4D). This indicates that high methylation in the Groups 2 and 3 of PHIM-CGIs may contribute to the lineage boundary of EXE and EPI (Yang et al., 2018), and their divergent development (Figure 4B). In contrast, compared with EPI, CGIs that are specifically highly methylated in the outgrowth derived from ICM cultured with FGF4 (termed as FGF4.out) are significantly enriched in the Groups 3–5 of PHIM-CGIs (Figure 4D). The Groups 3–5 of PHIM-CGIs are *de novo* highly methylated upon TSC formation (Figure 4C). Therefore, the high methylation in the Groups 3–5 of PHIM-CGIs likely plays a critical role in TSC formation and maintenance. Intriguingly, the GO analysis of these highly differentially methylated CGIs reveals that the Groups 3–5 of PHIM-CGIs are enriched for GO terms related to lineage differentiation, such as cell fate commitment, neuron fate commitment, appendage development, etc. (Figure 4E). This finding suggests that the high methylation in the Groups 3–5 of PHIM-CGIs may also create a barrier for lineage commitment to restrict embryonic development, and ensure TSC formation.

### Scriptaid Treatment Largely Rescues Abnormally High Methylation in the Derivation of Nuclear Transfer Trophoblast Stem Cells

**FIGURE 5:**
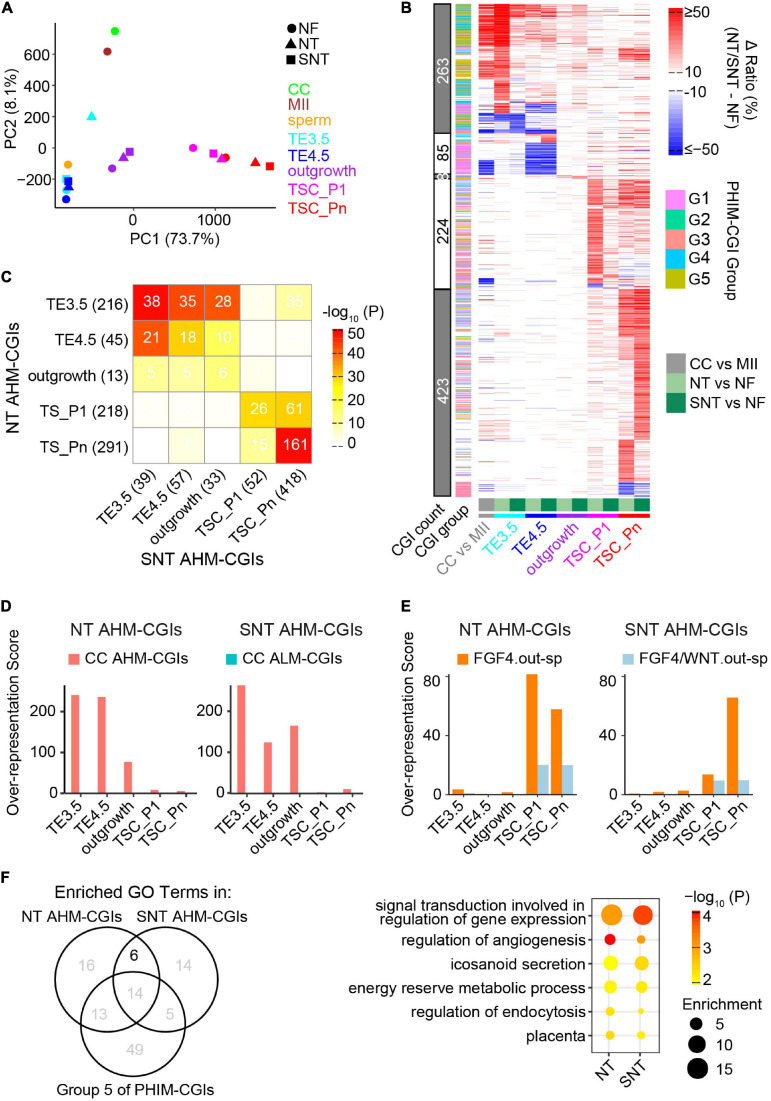
Abnormal methylation in the donor genome is a barrier to methylation remodeling during NT TSC derivation. **(A)** PCA results of CGI methylation during NF, NT, and SNT TSC derivation. CC (cumulus cell), MII oocyte, and sperm are also included for comparison. Their methylation data are from GSE56697 (Wang et al., 2014). **(B)** A heat map showing methylation difference of aberrantly highly methylated (AHM)- and aberrantly lowly methylated (ALM)-CGIs (Definitions are in section “Results”). **(C)** A heat map showing the intersection between NT and SNT AHM-CGIs. Filled colors indicate the significance of the intersection (hypergeometric test). Numbers indicate CGI count. **(D)** CC AHM-CGIs are enriched in early stages (TE3.5, TE4.5, and outgrowth) of NT/SNT TSC derivation. **(E)** “FGF4.out-sp” CGIs are specifically enriched in AHM-CGIs of NT and SNT TSC_P1, and TSC_Pn. “FGF4.out-sp” denotes the CGIs that are highly methylated in ICM treated with FGF4 but not in EXE and EPI. **(F)** Venn diagram showing the intersection the GO terms for which NT/SNT AHM-CGIs and the Group 5 of PHIM-CGIs (Figure 4C) are significantly enriched. The numbers indicate GO term count. The bubble plot showing the six GO terms specifically common to the GO terms for which NT/SNT AHM-CGIs are significantly enriched.

What is the scenario of DNA methylation dynamics during NT TSC derivation? Does HDAC inhibitor Scriptaid have a rescue effect in DNA methylation? To address this, we first compared the DNA methylation landscapes between NF, NT, and SNT TSC derivations. The results show that the global DNA methylation ratios remain low and are not significantly different between NF, NT, and SNT TSC derivations (Supplementary Figures 6A,B). However, PCA results of DNA methylation in CGIs revealed that NT TE3.5 is an outlier to NF and SNT TE3.5, although overall, there is a similar trajectory to the derivation path of NF, NT, and SNT TSCs. Besides, NT and SNT TSC_Pn deviate from NF TSC_Pn (Figure 5A).

We next identified aberrantly methylated CGIs during NT and SNT TSC derivation compared with the counterparts during NF TSC derivation. The CGIs whose DNA methylation ratios increase by more than 0.25 in NT or SNT TSC derivation are defined as AHM-CGIs. Similarly, the CGIs whose DNA methylation ratios decrease by more than 0.25 in NT or SNT TSC derivation are defined as aberrantly lowly methylated CGIs (ALM-CGIs). There are more than 200 AHM-CGIs in TE3.5, TSC_P1, and TSC_Pn and less than 50 AHM-CGIs in TE4.5 and outgrowth during NT TSC derivation. In contrast, only TSC_Pn exists in a large number of AHM-CGIs during SNT TSC derivation (Supplementary Figure 6C). TE3.5 AHM-CGIs are significantly enriched in the Groups 2 and 3 of PHIM-CGIs, while TSC_Pn AHM-CGIs are significantly enriched in Group 5 (Supplementary Figure 6D). Further analyses show that most AHM CGIs in NT TE3.5 are corrected in SNT TE3.5; so are AHM CGIs in NT TSC_P1 (Figure 5B). Quantification results reveal that only 38 out of 216 AHM CGIs in NT TE3.5 are not corrected in SNT TE3.5. Moreover, these 38 AHM CGIs account for 39 AHM CGIs in SNT TE3.5. Most of these 38 AHM CGIs remain uncorrected in SNT TE4.5 and outgrowth. Besides, more than half of AHM CGIs in NT TSC_Pn remain uncorrected in SNT TSC_Pn (Figure 5C). Surprisingly, when we replaced NT TE3.5 AHM-CGIs with the methylation ratios in NF TE3.5 and redid PCA, the results turn out that NF, NT, and SNT TE3.5 were clustered together. When we performed similar analysis for NT and SNT TSC_Pn AHM-CGIs, NF, NT, and SNT TSC_Pn were clustered closer (Supplementary Figure 6E). This indicates that correct DNA methylation is critical to TSC derivation. Scriptaid treatment can greatly rescue aberrant methylation, especially in TE3.5.

### Abnormal High DNA Methylation in Donor Nuclear Genome Contributes to Aberrantly Highly Methylated CGIs During Nuclear Transfer Trophoblast Stem Cell Derivation

It had been reported that epigenetic reprogramming is incomplete in NT. DNA methylation in many genomic regions of the donor cell are resistant to be reprogrammed even in early NT blastocysts (Gao R. et al., 2018). HDAC inhibitor treatment improves the donor epigenome reprograming (Jin et al., 2017). To explore how methylome of the donor cell (cumulus cell, CC) impacts the DNA methylome remodeling during NT TSC derivation, we identified AHM- and ALM-CGIs between CC and MII oocyte because CGIs lack methylation in sperm (Wang et al., 2014). More than half of AHM- and ALM-CGIs in NT and SNT TE3.5, TE4.5, and outgrowth are from the counterparts in CC while the majority of AHM- and ALM-CGIs in NT and SNT TSC_P1 and TSC_Pn are *de novo* ones (Figure 5B and Supplementary Figure 6C). Further analyses show that CC AHM- and ALM-CGIs are enriched in the counterparts in NT and SNT TE3.5, TE4.5, and outgrowth (Figure 5D and Supplementary Figure 6F). Contrarily, the specifically highly methylated CGIs in the outgrowth cultured with FGF4 are specifically enriched in AHM-CGIs of NT and SNT TSC_P1 and TSC_Pn (Figure 5E). Collectively, the aberrantly high DNA methylation in the donor genome failed to be remodeled and results in AHM- and ALM-CGIs during NT TSC derivation. Besides, FGF4 introduces abnormally high methylation in CGIs upon TSC formation and passaging.

### Functions of the Aberrantly Highly Methylated CGIs in Nuclear Transfer Trophoblast Stem Cell

Trophoblast stem cells are the resource library of cells for placenta development (Oda et al., 2006), and abnormal methylation may affect TSC differentiation. TSCs are maintained through passaging. Thus, it is important to understand the potential functions which TSC_Pn AHM-CGIs impact. Therefore, we performed GO analysis of AHM-CGIs of NT and SNT TSC_Pn. It is notable that AHM-CGIs of TSC_Pn largely intersected with Group 5 of PHIM-CGIs (Supplementary Figure 6D). The Group 5 of PHIM-CGIs became highly methylated upon TSC formation, while AHM-CGIs of NT and SNT TSC_Pn took place during passaging (Figure 4C and Supplementary Figure 6C). Therefore, we retained the common GO terms significantly enriched in AHM-CGIs of NT and SNT TSC_Pn, excluding those significantly enriched in the Group 5 of PHIM-CGIs, and obtained six GO terms (Figure 5F). Intriguingly, the six GO terms include signal transduction, energy reserve, angiogenesis, and placenta, which are all important to post-implantation development (Reynolds and Redmer, 2001). The CGIs related to the genes defining the six GO terms are significantly higher methylated in NT and SNT TSC_Pn than in NF TSC_Pn. Moreover, they remain low methylated post-implantation [e.g., EXE6.5, E10.5p (Legault et al., 2020), E15p (Decato et al., 2017), and placenta (Hon et al., 2013)] (Supplementary Figure 6G). Consistently, the 218 downregulated genes in NT + TSA placenta compared with IVF placenta have significantly higher methylation in both NT and SNT TSC_Pn than in NF TSC_Pn (Supplementary Figure 6H). Together, HDAC inhibitor treatment fails to correct many AHM-CGIs in NT TSC_Pn, which play a critical role in TSC differentiation and placenta development.

## Discussion

Trophoblast stem cells produce the cell source of trophoblasts and are important to placenta development. TSCs can be derived from blastocysts cultured with FGF4. Our study revealed that FGF4 treatment led to transcription and DNA methylation reprogramming that facilitates NF TSC derivation. However, there exist many variations in gene expressions and DNA methylation establishment during NT TSC derivation. The native DNA methylation landscape of the donor genome results in the aberrant methylation before NT TSC formation. In contrast, FGF4 treatment contributes to the aberrant methylation upon NT TSC formation and afterward. Most of the aberrant methylations are rescued during SNT TSC derivation. These findings will facilitate to improve NT and SNT TSC derivation.

DNA methylation is a key epigenetic factor regulating embryonic development. It has been reported that aberrant re-methylation impedes post-implantation of NT embryos (Gao R. et al., 2018). We found that the methylation profiles of NT and SNT TSC_P1 are closer to NF TSC_Pn than to NF TSC_P1. This implies that the earlier high methylation in the related CGIs accelerates the stem cell senescence process (Ohm and Baylin, 2007; Beerman et al., 2013) in NT and SNT TSCs. Besides, the aberrant high methylation in NT TSCs also disturbs gene imprinting. Recently, studies have proved that DNA methylation-independent H3K27me3 imprinting differentiates in embryonic and extra-embryonic cell lineage and is an epigenetic barrier impeding post-implantation development of NT embryos. Monoallelic imprinting gene (e.g., *Sfmbt2*) deletions in donor cells prevent the placental overgrowth defect and greatly improves fibroblast cloning efficiency (Inoue et al., 2017; Wang et al., 2020). Coincidently, our study showed that aberrant high methylation in the CGIs is associated with *Sfmbt2* in NT TSCs. This is consistent with the previous finding that NT placentas lose maternal H3K27me3 imprinting at the *Sfmbt2* loci (Matoba et al., 2018; Inoue et al., 2020; Wang et al., 2020). Of note, in addition to DNA methylation, there are other epigenetic factors that affect post-implantation development of NT embryos. For example, the native H3K9me3 landscape in the donor genome impedes the remodeling of chromatin state and 3D structure during NT embryo development (Chen et al., 2020; Yang et al., 2021). Therefore, DNA methylation alone has no or weak correlation with gene expressions during TSC derivation.

We identified a set of TFs comprising a tight interaction network that is important to TSC derivation. The network component TF ZFP281 has been reported as a protein factor that regulates the transcription programs of TSCs and early placenta development, which is sufficient to induce TSC-like cells (Ishiuchi et al., 2019). Consistently, the activity of this network is downregulated in NT TSC derivation but largely rescued in SNT TSC derivation. This further confirms the important functions of TF ZFP281 in TSC derivation. Intriguingly, ZFP281 also coordinates with TET1 and TET2 to establish and maintain primed pluripotency (Fidalgo et al., 2016). However, DNA methylation at the *Zfp281* locus remains a low level and has no correlation with its transcription. Therefore, the molecular basis underlying the transcription dynamics of *Zfp281* during TSC derivation remains unresolved. Besides, it is unclear whether ZFP281 is the core factor in the network. How does ZFP281 regulate the other network members comprising specific signaling pathways? To address these issues requires further study.

## Materials and Methods

### Mice

Mice were raised under SPF conditions under a 12-h light/dark cycle at 22 ± 2°C and with free access to standard mouse chow and tap water in the animal facility at Tongji University, Shanghai, China. We performed all mouse experiments according to the University of Health Guide for the Care and Use of Laboratory Animals.

### Blastocyst Collection

We obtained NF blastocysts by flushing on day 3.5 blastocysts from NF mice of B6D2F1 (8–10 weeks old) and then cultured blastocysts in G1 medium (Vitrolife, Göteborg, Sweden) with amino acids under 5% CO_2_ at 37°C.

### Somatic Cell Nuclear Transfer

We collected both oocytes and CCs from 8- to 10-week-old B6D2F1 female mice by superovulation. Superovulation was induced by sequentially injecting 7 IU of PMSG and 5 IU of hCG (San-Sheng Pharmaceutical, China) at an interval of 48 h. Then, cumulus–oocyte complexes were collected from oviducts 14 h after hCG injection and treated with hyaluronidase from bovine testes (Sigma, St. Louis, MO, United States) to obtain dissociated CCs and oocytes.

The oocytes were enucleated in a chamber containing oil-covered HCZB supplemented with 5 μg/ml of CB (Sigma) by Piezo-driven pipette (PrimeT 130 each) of an Olympus inverted microscope (Tokyo, Japan). The nuclei of donor CCs were transferred into enucleated oocytes by direct injection and activated through 5 h incubation in Ca^2+^-free CZB containing 1 mM SrCl_2_ and 5 μg/ml CB. The reconstructed embryos were thoroughly washed and cultured in G1 medium under 5% CO_2_ at 37°C.

For NT with the HDACi treatment, Scriptaid (Sigma, United States) was employed for a total of 10 h with a concentration of 5 nM by adding to the culture medium at the beginning of zygote activation.

### Derivation and Culture of Trophoblast Stem Cells

The derivation of TSCs was performed as published before (Gao H. et al., 2018). In brief, we transferred E4.5 blastocysts onto MMC-treated MEFs and first culture in TSC medium composed of 70% FCM, 30% TSM, and 1× F4H medium (TSM: RPMI1640 supplemented with 20% FBS, 1 mM sodium pyruvate, 100 μM β-mercaptoethanol, and 2 mM L-glutamine; 1× F4H: 25 ng/ml of FGF4, and 1 μg/ml of heparin). The time when the E3.5 blastocysts were placed into culture was designated as day 0. Once the embryos were attached, they would form an outgrowth on the second day, or an additional 1 to 2 days are required for attaching to occur. Thorough disaggregation of the blastocyst/TSC outgrowths by trypsin on days 5, 6, or 7 when the outgrowth is 800 to 1,000 μm in diameter was performed, and the resulting cell aggregates in fresh 70% FCM + 1.5× F4H medium were further cultured. Tight epithelial TSC colonies will become apparent 3 to 7 days after disaggregation is completed. The time when the TSC colonies appeared was designated as passage 1. When the TSC colonies appear overgrown or reach 80% confluency, TSCs were passaged with trypsin in 1× F4H medium. The culture of TSCs was gradually expanded every 4–6 days for several passages. The time when the virtually immortal TSC lines without significant differentiation were established was designated as passage n. Of note, all E3.5 and E4.5 blastocysts were cultured *in vitro* except that NF E3.5 blastocysts were developed in the uterus of B6D2F1 pregnant female mice.

### Sequencing Samples

We harvested samples: day 3.5 blastocysts TE (TE3.5), day 4.5 blastocysts (TE4.5), outgrowths (outgrowth), TSCs passage 1 (TSC_P1), and TSCs passages 3–4 (TSC_Pn) from NF, somatic cell NT, and HDACi Scriptaid-treated NT (SNT) blastocysts. For TE isolation, we treated blastocysts in Ca^2+^-free CZB for 20 min and separated the junctions of TE and ICM by multiple pipetting using a pipette with a diameter of 40–60 μm. Besides, the zona pellucida were removed from E3.5 blastocysts by 0.5% pronase. To collect cells of outgrowth, TSC_P1, and TSC_Pn, we washed the collected cells three times using DPBS and then disaggregated the cells using 0.05% Trypsin-EDTA.

### Reduced Representation Bisulfite Sequencing and RNA-seq

We performed RRBS and RNA-seq following a previously published study (Liu et al., 2016). Briefly, we washed cells three times in 0.5% BSA-PBS (Sigma) solution and hereafter transferred cells into a lysate buffer using a mouth pipette.

For the RRBS sequencing library, we digested nuclear proteins and extracted the DNA from the nuclei of the samples. Then, we added unmethylated lambda DNA (Fermentas) and took a one-tube reaction, treating the DNA with MspI digestion (Fermentas, United States), end repair, dA tailing, adaptor ligation, and bisulfite conversion using a MethylCode Bisulfite Conversion Kit (Invitrogen, United States, MECOV-50). We purified the converted DNA libraries by Agencourt AMPure XP beads (Beckman A63881, United States) and amplified the DNA using a two-round PCR enrichment. Only 200- to 500-bp DNA fragments were retained for sequencing.

For RNA-seq, we performed reverse transcription directly on the cytoplasmic lysate and used terminal deoxynucleotidyl transferase to add a poly(A) tail to the 3′ end of the first-strand cDNAs. We amplified the total cDNA library by 18–20 cycles. Afterward, we fragmented the amplified cDNA by Covaris sonicator (Covaris S220, United States) and used the TruSeq Library Prep Pooling kit (Illumina 15042173, United States) to generate the RNA sequence libraries.

We performed paired-end 125- or 100-bp sequencing on HiSeq 2000 or 2500 (Illumina) at the Peking University and Berry Genomics Corporation.

### Reduced Representation Bisulfite Sequencing and RNA-seq Data Processing

Reduced representation bisulfite sequencing reads were first processed using trim_galore (0.4.2) to trim adaptor and low-quality reads by parameters -fastqc -illumina -rrbs -paired, and then aligned to a combined genome with mm 10 and lambda sequence using bsmap (1.3.2) (Xi and Li, 2009) by parameters -D C-CGG -s 12 -v 0.1 -R -r 0. The methylation level of each CpG was estimated using mcall (1.3.2) (Sun et al., 2014) by default parameters. Only the CpGs with coverage >4 in replicates were retained for later analysis.

The adaptors and low-quality reads were removed from the RNA-seq data using cutadapt (1.11) (Martin, 2011) by parameters -a AGATCGGAAGAGCACACGTCTGAACTCCAGTCAC -A AGATCGGAAGAGCGTCGTGTAGGGAAAGAGTGTAGATC TCGGTGGTCGCCGTATCATT -m 50 -q 20. Then the RNA-seq reads were aligned to mm 10 transcript genome using STAR (020201) (Dobin et al., 2013) by parameters -readFilesCommand zcat -runThreadN 8 -outFilterMismatchNmax 3. The uniquely mapped reads were subsequently assembled into known transcripts (iGenome mm10) with featureCounts (v1.6.1) (Liao et al., 2014).

### Reduced Representation Bisulfite Sequencing Data Analysis

CpG density is calculated 300 bp around the center CpG site using “linear” weighting by compEpiTools (1.12.0) (Kishore et al., 2015). HCP, ICP, and LCP were defined as previously published (Weber et al., 2007). For CGI analysis, we collected mm10 CGI table from UCSC table browser and only retained the CGIs with detected CpG > 4 among all samples. We calculated CGI methylation using the mean ratio of all CpGs contained. We used a robust cutoff with methylation difference >25% and *p*-value of Fisher’s test <0.05 to define the differential CGIs. Highly methylated CGIs have a methylation ratio >25%. Of all genes whose transcript start sites (TSSs) are located within 5 kb of a CGI, the closest gene is associated with the CGI.

### Normalized Gene Expression and Differentially Expressed Genes

We calculated log_2_ (RPM+1) as the normalized expression using edgeR (3.20.9) (Robinson et al., 2009) and retained the genes with normalized expression larger than one at least in one sample for further analysis. We identified the genes that were differentially expressed (DE-genes) using DESeq2 (1.18.1) (Love et al., 2014). We required the adjusted *p*-value to be <0.01 and the log_2_ fold change to be >2. For public data, we defined DEGs using a cutoff of FDR <0.01 and log_2_ fold change >1.

### Motif Analysis

We performed motif analysis for the 748 vertebrate motifs in the JASPAR_CORE_2018_vertebrate database (Khan et al., 2018) using findMontifs.pl from HOMER (v4.10) by searching motifs in the regions that are −500 to +300 bp relative to the TSSs.

### Statistical Test

For statistical comparison between two samples, we performed paired *t*-test and used “Holm” to adjust *p*-values. As for the intersection of two sets, we performed hypergeometric test. We weighed the enrichment of the intersection using the representation factor (RF), which was calculated using real observation/expected observation. We also defined the Over-representation Score to count the enrichment and *p*-value together by calculating log_2_(RF) × log_10_(*p*-value). Besides, we used edgeR to test the significance of gene expression differences from public data.

### Gene Ontology Analysis

We performed enrichment analysis of GO terms and tissue pattern genes collected from PaGenBase (Pan et al., 2013) using metascape (Zhou et al., 2019). The enrichment was calculated by the (ratio of term genes in hit/ratio of all term genes in total). Only the results with *p* < 0.01 were retained as enriched terms.

### Pseudotime Inference

We inferred the pseudotime of a replicate using *T* = Σ*_*i*_*^75^ Exp*_*i*_* × λ*_*i*_*. Exp*_*i*_* indicated the normalized expression of the *i*-th TF in the replicate. λ indicated the coefficients of the TFs. We used the corresponding PC1 loadings of PCA analysis on NF samples as λ. We also randomly sampled the same number of TFs and did pseudotime inference for 5,000 iterations to get a mean result.

### ChIP-seq Analysis

We aligned public ChIP-seq data to mm10 using bwa (0.7.12-r1039) (Li and Durbin, 2009) and then we discarded PCR duplicates and multiply-mapped reads. We performed peak calling using macs2 (2.1.1.20160309) (Zhang et al., 2008) with parameters -g mm -B -SPMR -nomodel -shift 37 -extsize 73 and transformed the fragment pileup and control lambda to bigwig format. Then, we computed the ChIP-seq signal around TSSs using computeMatrix from deeptools (2.5.7) (Ramírez et al., 2016).

## Data Availability Statement

The datasets presented in this study can be found in online repositories. The names of the repository/repositories and accession number(s) can be found in the article/Supplementary Material.

## Ethics Statement

The animal study was reviewed and approved by the Biological Research Ethics Committee of Tongji University.

## Author Contributions

CJ and SG conceived and designed the experiments, and wrote the manuscript. RG constructed the TSC lines and sequencing libraries, and prepared the samples. WL did the cloning. XK and YZ helped with the experiments. JS and WZ did most of the data analyses with the help of LW, ZL, ZZ, and JX. All authors read and approved the final manuscript.

## Conflict of Interest

The authors declare that the research was conducted in the absence of any commercial or financial relationships that could be construed as a potential conflict of interest.
