# Supplementary material for: Differential Transcriptomes and Methylomes of Trophoblast Stem Cells From Naturally-Fertilized and Somatic Cell Nuclear-Transferred Embryos
Source: Front Cell Dev Biol. 2021 Apr 1;9:664178. doi: 10.3389/fcell.2021.664178 (PMC8047118; doi:10.3389/fcell.2021.664178)
Supplement: Supplementary file 1 [file Table_1.DOCX]

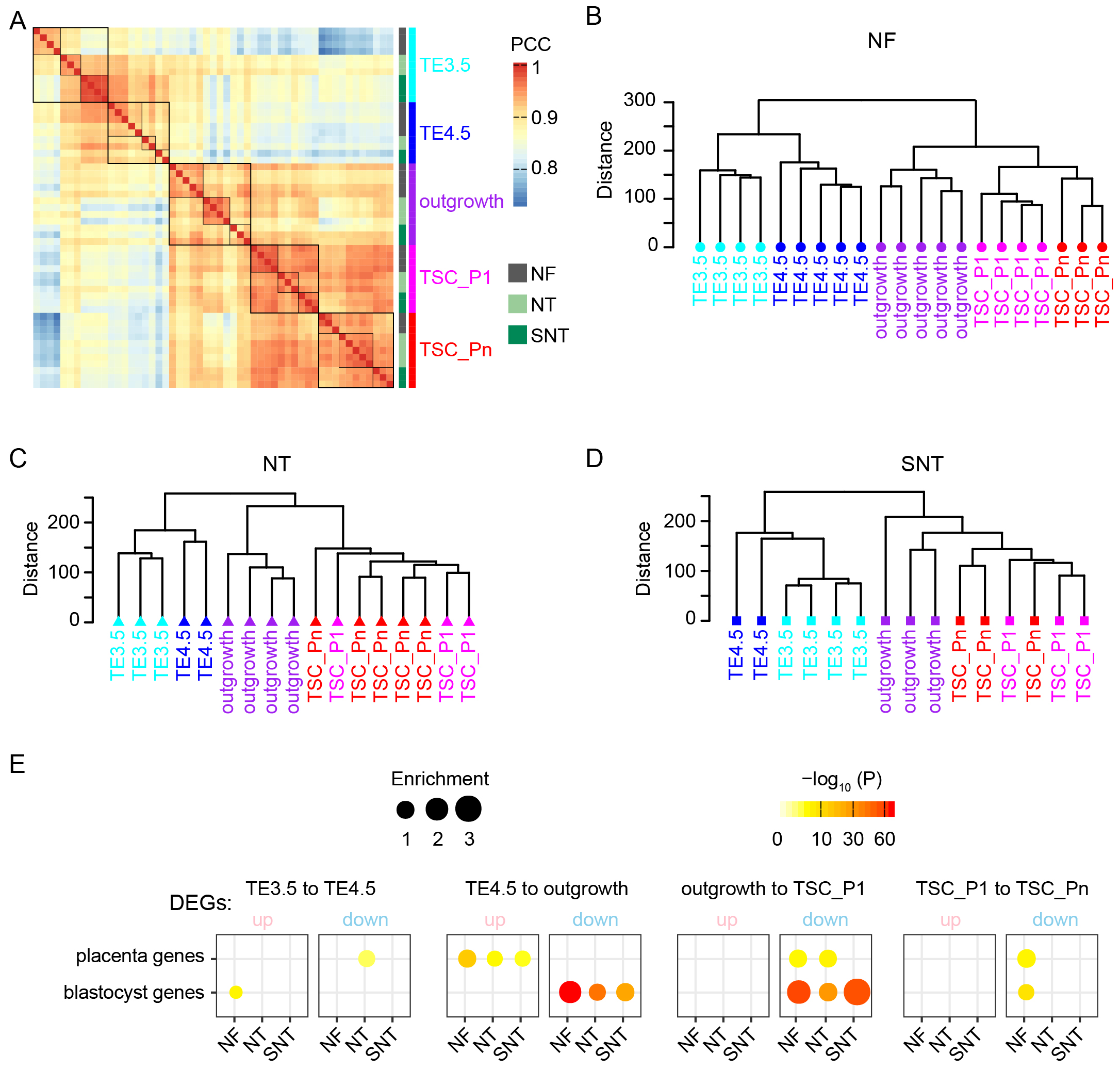


**SUPPLEMENTARY FIGURE S1** | Characterizing gene expression dynamics during NF, NT, and SNT TSC derivation

**(A)** A heat map showing Pearson’s correlation coefficient of normalized gene expressions between all replicates.

**(B)**, C, D Dendrograms showing clustering of normalized gene expressions for replicates in different groups. B, NF; C, NT; D, SNT.

**(E)** Bubble plots showing the enrichment of placenta- and blastocyst-specific genes in the DEGs between adjacent stages during TSC derivation (Figure 1D). (hypergeometric test)


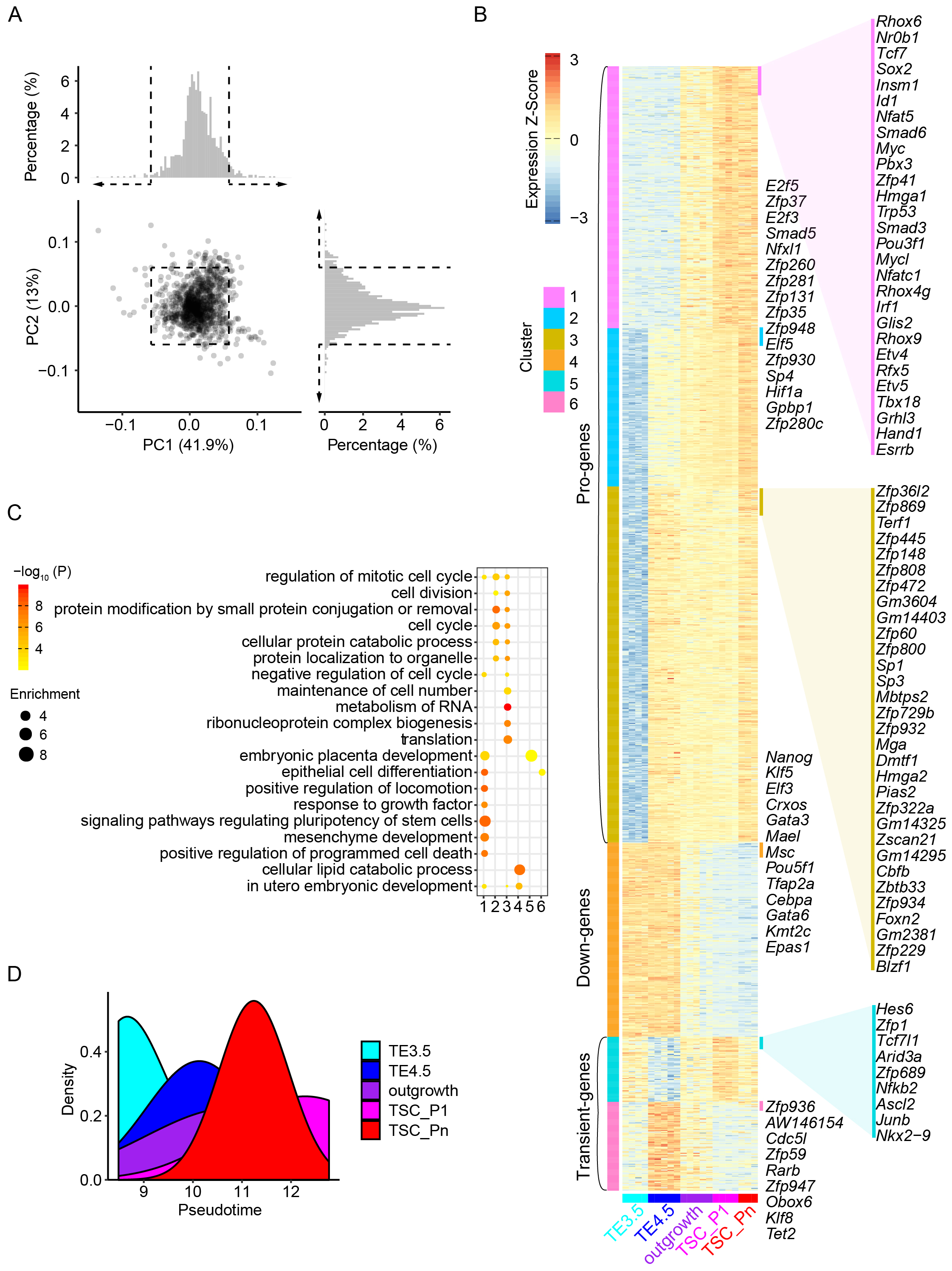


**SUPPLEMENTARY FIGURE S2** | Profiling of the key dynamically expressed genes during the derivation of NF TSCs

**(A)** A scatterplot showing PC1 and PC2 loadings of genes. The dashed lines show cutoffs of top 5% of absolute values of PC1 and PC2 loadings. The genes beyond the rectangle are defined as dynamically expressed genes.

**(B)** A heat map showing gene expression patterns of the dynamically expressed genes. The unsupervised hierarchical clustering of their expression resulted in 6 clusters.

**(C)** A bubble plot showing gene ontology terms (biological processes) for which the six gene clusters in (B) are enriched. The sizes of the dots represent enrichment score and colors represent enrichment significance (p-value of hypergeometric test).

**(D)** The disordered pseudotime of NF TSC derivation inferred from the transcriptomes of the randomly selected 75 TFs (5000 iterations).


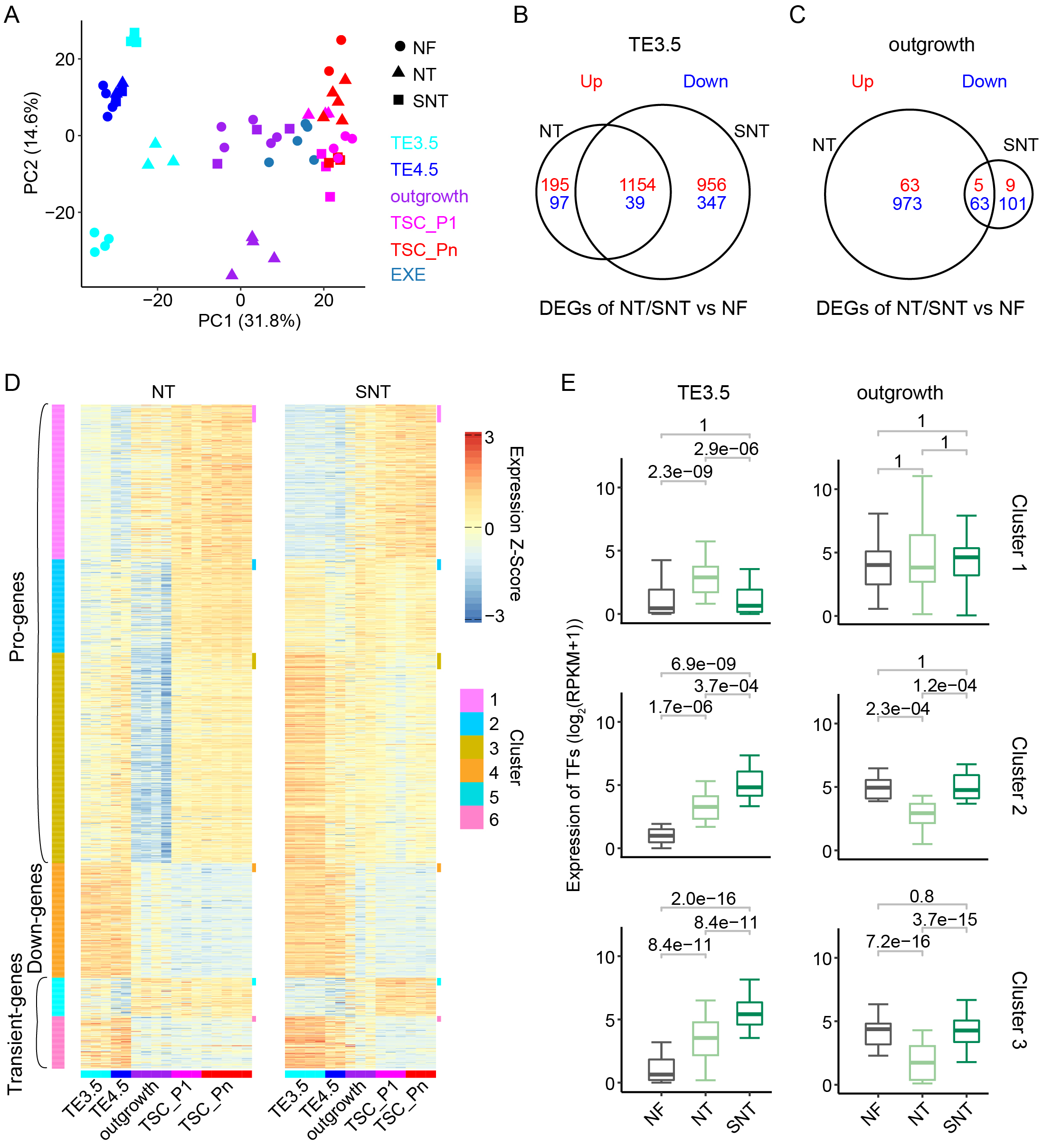


**SUPPLEMENTARY FIGURE S3** | The disturbing expression dynamics during NT and SNT TSC derivation

**(A)** Principal component analysis (PCA) of all the transcriptomic data in the derivation process of TSCs.

**(B)** Venn diagrams showing the intersection of DEGs between NT/SNT and NF TE3.5. Red numbers are up-regulated DEG count. Blue numbers are down-regulated DEG count.

**(C)** Venn diagrams showing the intersection of DEGs between NT/SNT and NF outgrowth. Red numbers are up-regulated DEG count. Blue numbers are down-regulated DEG count.

**(D)** Heatmaps showing the expression patterns in NT and SNT TSC derivation of the key dynamically expressed genes identified in NF TSC derivation (Supplementary Figure S2B). Genes are sorted as in Supplementary Figure S2B.

**(E)** Boxplots showing normalized expressions of the TFs in Cluster 1-3 in TE3.5 (left) and outgrowth (right) during TSC derivation.


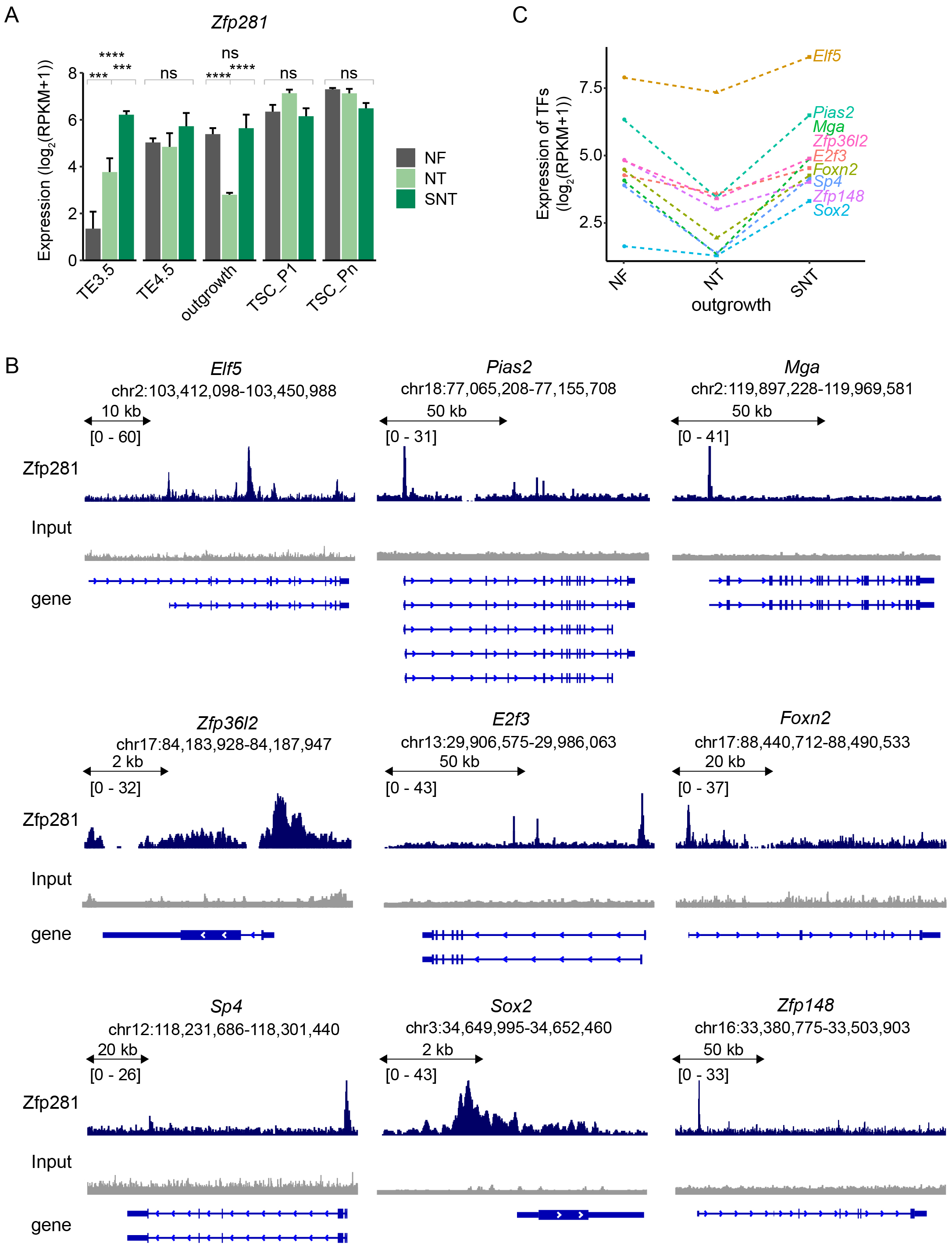


**SUPPLEMENTARY FIGURE S4** | Down-regulated expression of Zfp281 in NT outgrowth

**(A)** Barplots showing the normalized expression of Zfp281 during TSC derivation. Error bars represent 95% confidence intervals. ( ****: p.adjust <= 0.0001, ***: p.adjust <= 0.001, ns: not significant, DESeq2 for Statistical test (Love et al., 2014)).

**(B)** Line plots showing the down-regulated expression of 9 TFs in NT outgrowth whose promoter regions are bound by ZFP281. The 9 TFs are Clusters 1-3 (Supplementary Figure S2B)

**(C)** IGV browsers showing ZFP281 binding in the promoters of the 9 TFs in (B).


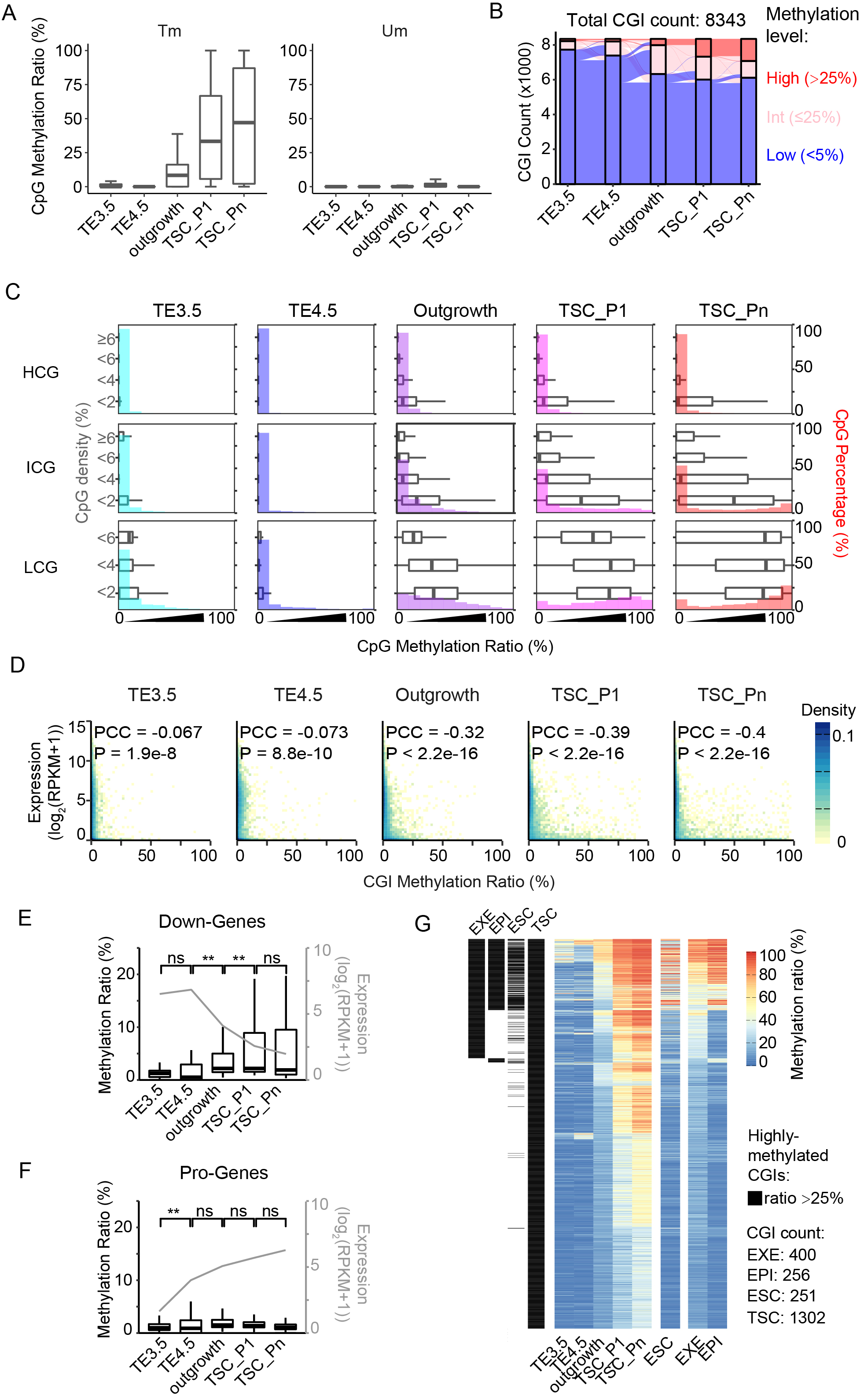


**SUPPLEMENTARY FIGURE S5** | Profiling of DNA methylation during the Derivation of TSCs.

**(A)** CpG methylation ratio in the two sets of lociidentified in the previous study (Oda et al., 2009). Tm: TSC-specific methylated sites, Um: TSC-unmethylated sites. The horizontal lines denote medians. Edges refer to the 25^th^ and 75^th^ percentiles, whiskers are the 25^th^ percentile -1.5*IQR (the interquartile range) or smallest observation and 75th percentile +1.5*IQR (the interquartile range) or largest observation, respectively.

**(B)** Alluvial plot showing CGI methylation dynamics during the derivation of TSCs.

**(C)** CpG methylation levels in the categorized promoters HCPs, ICPs and LCPs. The histograms (right axis) showing the distribution of methylation levels binned by 0.10 (bottom axis). The boxplots showing methylation levels of promoters conditional on local CpG density (left axis). The vertical lines denote medians.

**(D)** Scatterplots showing the correlation between CGI methylation and related gene expression.

**(E,F)** DNA methylation has no correlation with Pro-genes (E) expression and negative correlation with Down-genes expression (F). The curve plot showing expression of Pro-genes and Down-genes during TSC derivation. The boxplots showing DNA methylation levels of CGIs associated with Pro-genes and Down-genes. The horizontal lines denote medians. (**: p value <= 0.01, ns: not significant, paired t-test and holm for adjust.)

**(G)** A heat map showing methylation changes of all highly-methylated CGIs (> 0.25) during NF TSC derivation. The majority of these CGIs are *de novo* highly methylated upon TSC formation. The black lines indicate the highly-methylated CGIs in each sample. The numbers indicate their counts. The RRBS data of ESCs are from GSE47343 (Guo et al., 2013).


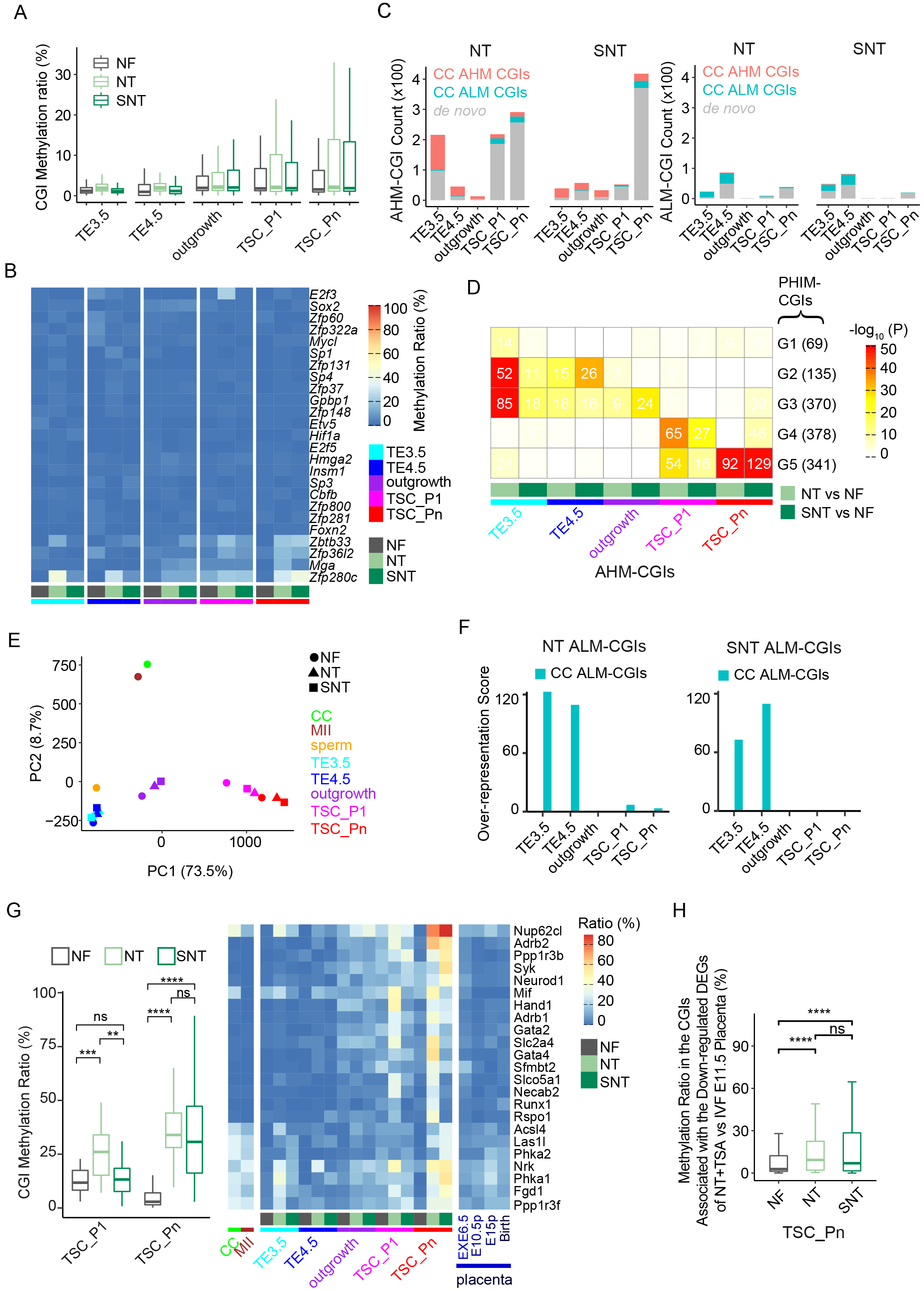


**SUPPLEMENTARY FIGURE S6** | Comparison of DNA methylation dynamics between NF, NT and SNT TSC derivation

**(A)** Boxplots showing CGI methylation ratios. The horizontal lines indicate medians. Edges refer to the 25^th^ and 75^th^ percentiles, whiskers the 25^th^ percentile -1.5*IQR (the interquartile range) or smallest observation and 75th percentile +1.5*IQR (the interquartile range) or largest observation, respectively.

**(B)** A heat map showing methylation ratios of the CGIs associated with the TFs in the Pro-genes (Figure 2B) which are significantly down-regulated in NT outgrowth. This indicates that DNA methylation has no correlation with gene expression.

**(C)** Bar plots showing the counts of AHM- and ALM-CGIs categorized by the methylation ratio difference between CC and MII oocyte. AHM-CGIs (aberrantly highly methylated CGIs) denote the CGIs whose methylation ratios are 25% higher in NT or SNT than NF while ALM-CGIs (aberrantly lowly methylated CGIs) are opposite. CC AHM-CGIs denote CGIs whose methylation ratios are 25% higher in CC than MII oocyte. CC ALM-CGIs are opposite. *de novo* denotes CGIs whose methylation ratio difference between CC and MII oocyte is less than 25%. That is, these NT and SNT AHM- and ALM-CGIs are *de novo* ones, not inherited from CC AHM- or ALM-CGIs.

**(D)** A heat map showing the intersection between NT/SNT AHM-CGIs and the PHIM-CGIs (Figure 4C). Filled colors indicate the significance of the intersection (hypergeometric test). Numbers indicate CGI count.

**(E)** Similar PCA of CGI methylation during NF, NT and SNT TSC derivation, CC (cumulus cell), MII oocyte and sperm as in Figure 5A except that methylation levels of NT TE3.5 AHM-CGIs are replaced with those of NF TE3.5, and methylation levels of NT/SNT TS_Pn AHM-CGIs are replaced with those of NF TS_Pn. The methylation data of CC, MII oocyte and sperm are from GSE56697 (Wang et al., 2014).

**(F)** CC ALM-CGIs are enriched in early stages (TE3.5 and TE4.5) of NT/SNT TSC derivation.

**(G)** The left boxplots showing the methylation levels of CGIs associated with the genes defining the 6 GO terms in Figure 5F. The right heat map showing the methylation level of these CGIs during the derivation of TSCs and post-implantation placentas. The methylation data of post-implantation placentas are from GSE95610 (Legault et al., 2020), GSE84350 (Decato et al., 2017), and GSE42836 (Hon et al., 2013). (****: p.adjust <= 0.0001, ***: p.adjust <= 0.001, **: p.adjust <= 0.01, ns: p.adjust > 0.05, paired t-test and holm for adjust.)

**(H)** Boxplot showing the TSC_Pn methylation levels of CGIs associated with the down-regulated DEGs of NT+TSA vs IVF E11.5 placentas (Inoue et al., 2020). TSA, trichostatin A, is an HDAC inhibitor.
